# Effects of stream grazers with different functional traits on the spatial heterogeneity of periphyton mats

**DOI:** 10.7717/peerj.6747

**Published:** 2019-06-05

**Authors:** Izumi Katano, Hideyuki Doi

**Affiliations:** 1 Graduate School of Humanities and Sciences, Nara Women’s University, Nara, Japan; 2 KYOUSEI Science Center for Life and Nature, Nara Women’s University, Nara, Japan; 3 Graduate School of Simulation Studies, University of Hyogo, Kobe, Japan

**Keywords:** Heterogeneity, Enclosure experiment, Stream grazer, Caddisfly, Mayfly

## Abstract

**Background:**

Grazing activity on periphytic mats determines mat structure and spatial heterogeneity. Spatial complexity in stream periphyton is highly divergent and may depend on the functional traits of the different primary consumers species (i.e., grazers) such as mouthpart morphology, feeding behavior, and feeding activity. We evaluated the effect of grazing by three species having different functional traits on periphytic mat structure with a focus on mohthpart morphology.

**Methods:**

An enclosure experiment was conducted in a stream located in the Nara Prefecture of Japan using two caddisflies with scraping mouthparts, *Micrasema quadriloba* and *Glossosoma*, and one mayfly, *Epeorus*, with brushing mouthparts. The spatial heterogeneity of chlorophyll *a*(Chl *a*) was evaluated, and the periphytic mat was analyzed using scanning electron microscopy (SEM) after a 12-d feeding experiment.

**Results:**

Our results showed the differences in the spatial heterogeneity of the periphytic mats, such as differences in Chl *a* levels, grazed by the different grazing species. The strongest effect on the spatial heterogeneity and periphytic abundance was observed for *Micrasema quadriloba*, a caddisfly species with scraping mouthparts. *Epeorus* mayfly, with brushing mouthparts and high-mobility behavior, produced the weakest effect on spatial heterogeneity. *Glossosoma* caddisflies had moderate effects on periphytic spatial heterogeneity and abundance. Our results suggest that differences in grazing effects are largely dependent on grazer mouthparts and behavior.

## Introduction

Periphytic algae are the most important primary producers in stream ecosystems (Minshall, 1978; Vannote et al., 1980; Lange et al., 2011). Grazer-periphyton interactions in stream environments have been used as model systems to understand more general producer-herbivore interactions (Hart & Robinson, 1990; Feminella & Hawkins, 1995; Doi, Katano & Kikuchi, 2006; Doi & Katano, 2008; Katano & Doi, 2014). The influence of resources and grazing on the composition of periphyton assemblages can be assessed both taxonomically and with regard to functional groups (Passy, 2008), two parameters that can be affected by light, nutrients, and grazing regimes (Johnson, Tuchman & Peterson, 1997; Hollingsworth & Vis, 2010; Lange et al., 2011).

The impact of grazing on the spatial complexity of periphyton has been shown to vary widely depending on grazer functional traits, including mouthpart morphology, aggregation behavior, and grazer activity (Liess & Hillebrand, 2004; Wellnitz & Poff, 2006). Grazing on periphytic mats results in a feeding trace that may change mat structure and heterogeneity (Pringle, 1990; Palmer, 1995; Wellnitz & Poff, 2006). Periphytic mat heterogeneity in turn may influence the fitness of grazer species by affecting their growth rates (Pringle, 1990; Palmer, 1995).

Insect grazers have different mouthpart morphologies and the impact of their feeding on periphytic mat structure has typically been evaluated by taking into account their mouthpart morphologies (Karouna & Fuller, 1992; Merritt & Cummins, 1996). However, other functional traits, such as feeding behavior, may also be important (Karouna & Fuller, 1992; Arens, 1994; Wellnitz & Ward, 2000). Therefore, to better understand the effects of grazing on periphytic mat structure, the effects of other functional traits must be evaluated.

Although the effect of grazers’ feeding traits on periphyton has been previously studied using the larvae of caddisflies and other insects (Karouna & Fuller, 1992; Arens, 1994), the effects on periphytic heterogeneity and the micro-structure of periphytic mats remains largely unknown. Investigating these effects of feeding traits is important for understanding how stream grazers shape the spatial structure of periphyton. Therefore, the aim of this study was to assess the effect of grazing by species with different feeding traits on the structure of periphytic mats, focusing on mouthpart morphology, body mass, and speed of movement, To address this aim, we conducted an enclosure experiment with three grazer species having different mouthpart morphologies, two with scraping mouthparts and one with brushing morphology. We also measured the spatial heterogeneity of chlorophyll (Chl *a*) and analyzed periphytic mat structure using scanning electron microscopy (SEM).

## Materials & Methods

### Study species

The study organisms were two species of case-bearing caddisflies (*Glossosoma* sp. and *Micrasema quadriloba*, Trichoptera) and one species of mayfly (*Epeorus* sp., Ephemeroptera). *Glossosoma* sp. and *M. quadriloba* are low-mobility grazers widely distributed in the mountain streams of central Japan (Katano et al., 2005; Katano et al., 2007) *Epeorus* sp. is a high-mobility species broadly distributed in the streams of central Honshu, Japan.

### Field sampling

On March 12, 2004, we investigated the natural larval densities of *M. quadriloba*, *Glossosoma* sp., and *Epeorus* sp. in the Shigo-gawa stream (stream width: 2–18 m, mean gradient: 2.2%) in Nara Prefecture, Japan (34°22′66″N, 136°01′00″E). Field sampling was conducted independently from that of Katano et al. (2005) and Katano et al. (2007). Permission was not required for invertebrate sampling according to Japanese law, but our field sampling was approved by the Higashi-yoshino fishermen’s cooperative. A 50 m riffle was established as the study reach. Within this reach, 32 cobbles (particle size: 64–256 mm) were randomly collected. Immediately after collection, the samples were fixed with a 4% formaldehyde solution and these species were identified using the taxonomic keys in Kawai (1985) in the laboratory.

### Enclosure experiment

**Figure 1:**
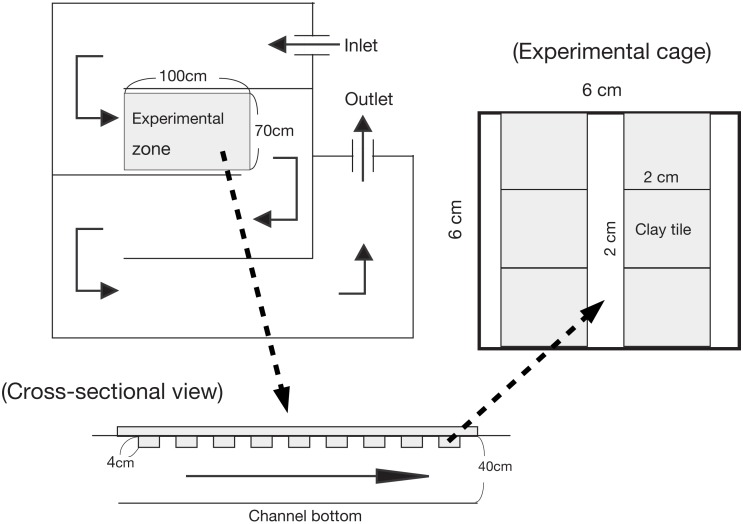
Illustration of the experimental channel and cage setup in this study. Water input was directly connected to a tributary of the Shigo-gawa stream.

For the enclosure experiment, an non-linear experimental channel was set up that was 10 m in total length, 0.70 m wide, and 0.40 m deep (see Katano et al., 2007; Fig. 1). The experimental channel was located near a small tributary of the Shigo-gawa stream, and the water was collected directly from the tributary. An experimental zone within the channel (1 m long; 7,000 cm^−2^ area) was established 2.5 m downstream of the water inlet. A polystyrene foam plate (2 cm thick) was set as a mooring float. The enclosures were 24 experimental cages measuring 6 × 6 × 6 cm and constructed of 0.2 mm nylon mesh, as used in Katano et al. (2007; Fig. 1). To minimize any reduction in periphyton productivity, the mooring float was manually cleaned once a day during the experimental period to prevent clogging by leaf litter.

The enclosure experiment in the channel was performed from March 16 to 27, 2004 (12 d). Six clay tiles (2 × 2 × 1 cm) with periphyton were placed in each cage and incubated in the channel for two months (Fig. 1). The ash-free dry weight (AFDW) was 2.8 ± 0.27 mg cm^−2^ (mean ± 1 SD, *N* = 4), and the chlorophyll *a* content was 10.1 ± 2.4 mg cm^−2^. The clay tiles measured 4.5 × 4.5 × 0.4 cm for Chl *a* measurement (48 tiles), and 2.25 × 2.25 × 0.4 cm for SEM (12 tiles). For Chl *a* measurement, the study cages were randomly divided into the following four treatments; control (without larvae), *M. quadriloba* with 23 larvae, *Glossosoma* sp. with three larvae, and *Epeorus* sp. with three larvae. Each treatment was replicated six times.

### Examination of grazer mouthparts and periphyton using SEM

We analyzed grazer mouthpart morphology of fourth instar larvae using scanning electron microscopy (SEM, × 10,000, Hitachi S-3000). For SEM observation, the individual larvae were dehydrated via an ethanol series (30, 50, 70, 90, 95, and 100%), 10 min at each concentration. The dehydrated sample was soaked with 100% isoamyl acetate for 1 h at room temperature, dried with CO_2_ using a critical point dryer (HPC-2 Critical Point Dryer; Hitachi, Tokyo, Japan), and sputter-coated with gold. The SEM images for periphytic mat on clay tiles were prepared and examined in a similar manner.

### Measurement of periphyton Chl *a*

**Figure 2:**
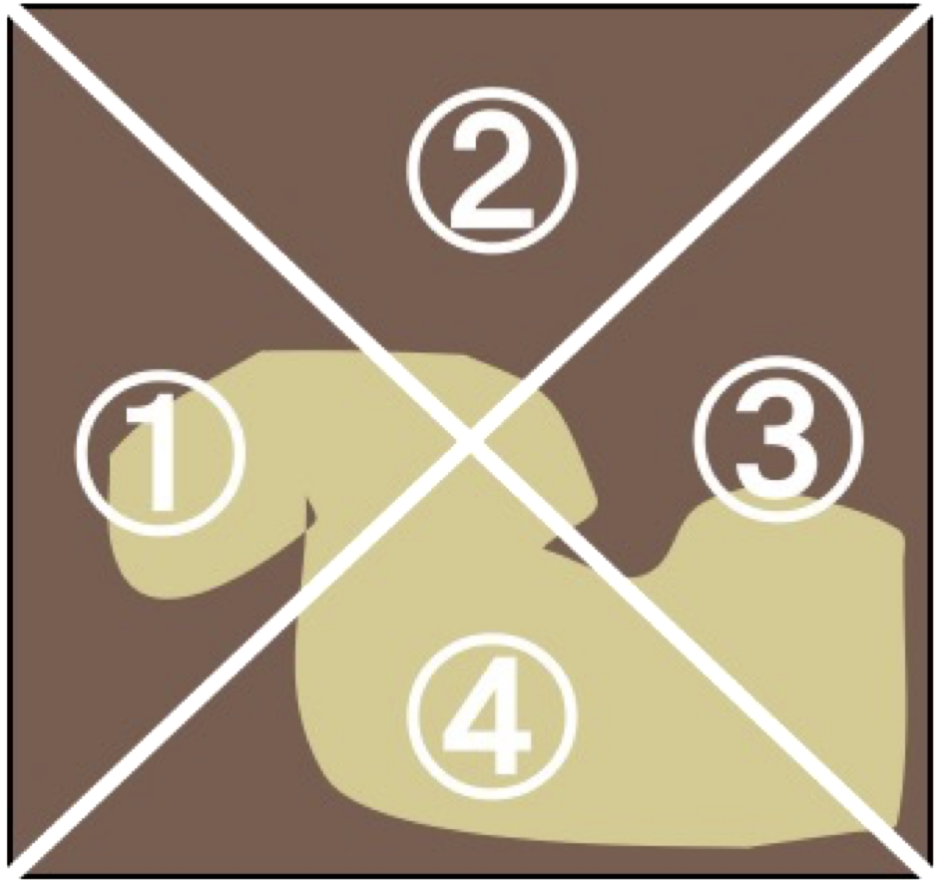
Illustrations of the measurements of periphyton chlorophyll *a* coefficients of variation (CV).

The abundance of microalgal cells in each treatment was estimated by measuring the Chl *a* concentration. The Chl *a* was measured according to the UNESCO method (1966), followed by assessment of the abundance of microalgae in the experimental tile. At the end of the experiment, periphyton from each tile (*N* = 12 for each treatment) were removed using a toothbrush and rinsed into a 100-mL container with tap water. The samples was then filtered through a glass filter (GA-100; Toyo-roshi Co., Tokyo, Japan; pore size, 1 µm). To extract the chlorophyll, the filter was cut into small pieces and placed into a vial, containing 20 mL of 99.5% ethanol. Vials were placed in the dark at 4 °C for 24 h. Following this, the absorbance spectra of the extracted pigments were measured at 480, 630, 645, 665, and 750 nm using a MPS-2000 spectrophotometer (Shimadzu Co., Japan). For six tiles of each treatment, the coefficient of variation in Chl *a* content was calculated from the Chl *a* content in four separate areas (zones 1–4 in Fig. 2). For six tiles of each treatment, the Chl *a* content was measured in both the grazed and non-grazed areas to the ratios of Chl *a* content in grazed/total periphyton area, and also we examined grazed area (cm^2^) by photograph of the tile using ImageJ software ver. 1.31.

### Measurement of grazer body mass, movement rate, and growth

Body mass of the species collected during field sampling were measured to the nearest 0.01 mg using an electronic balance (CP225D; Sartorius Co, Arvada, CO, USA). Six individuals of each species were measured. The movement rate of individuals was measured by recording the time in took to move across cage enclosure tiles from edge to edge, Movement rate were recorded on the first day of experiments (*N* = 6 for each species).

To estimate grazer growth rates, the initial and dry body weights of larvae were measured before and after the experiment. Twelve larvae from each species were collected before the experiment began and dried at 55 °C for 24 h. The larvae were then weighed, and the dry weight per larva was calculated for each species. At the end of the experiment, all larvae were collected from cage enclosures, and preserved in 4% formaldehyde. These larvae were then dried and weighed as described above. The daily relative growth rates for each species (RGR) was calculated as follows:

\begin{eqnarray*}& & \mathrm{RGR}=[(\mathrm{final~ dry~ weight})-(\mathrm{initial~ dry~ weight})]/12(\mathrm{d}). \end{eqnarray*}

### Statistical analysis

Differences among treatments were tested for significance using one-way analysis of variance (ANOVA), and the post-hoc multiple comparisons were performed using Tukey’s test. For all statistical analyses, *α* = 0.05 was used as the significance criterion. Preliminary Shapiro–Wilk tests for normality showed that the coefficients of variation (CV) of Chl *a* were not normally distributed (*P* > 0.05); therefore, these variables were log-transformed. All statistical analyses were performed using R version 3.3.3 (R Core Team, 2017).

## Results

**Figure 3:**
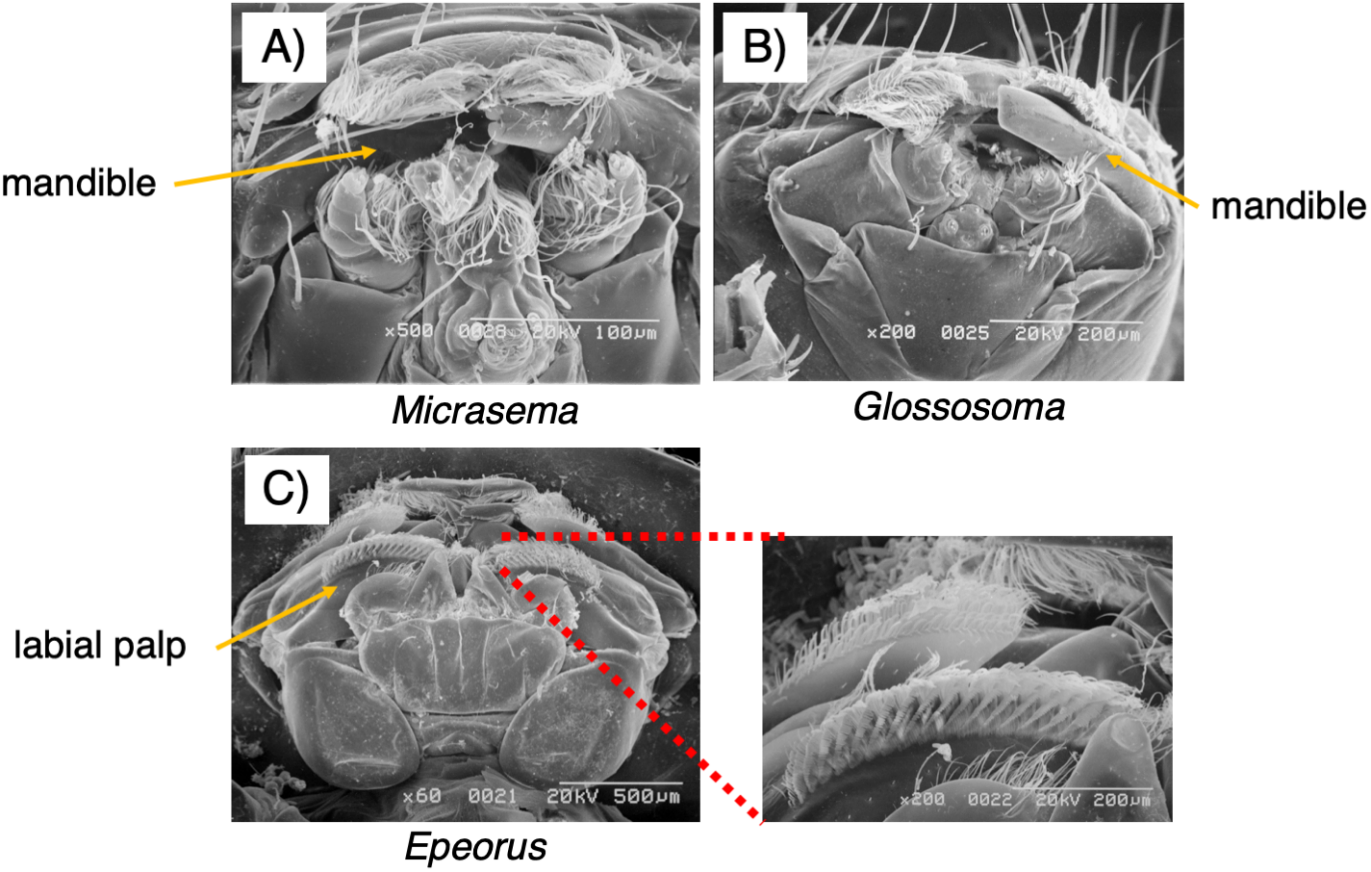
Scanning electron microscopy (SEM) images of the mouthpart morphologies of the three grazer species used for this study; (A) *Micrasema quadriloba*, (B) *Glossosoma* sp., and (C) *Epeorus* sp. Magnitude and scale are shown in the images.

As seen in Fig. 3, the mouthpart morphology of each grazer species was distinctive, and SEM confirmed that the *Glossosoma* sp. and *M. quadriloba* were scrapers, and *Epeorus* sp. was a brusher. Body mass and movement rates differed among species (Table 1), with *Epeorus* having the highest values (ANOVA; *F* = 45.8 and 6.63, *P* = 0.00001, Tukey; *P* = 0.0001, and <0.018, respectively). Growth rates showed large variation and were nott significantly different among the species (Table 1, ANOVA; *F* = 1.61, *P* = 0.232), possibly due to the relatively short duration of the experiment.

**Table 1:** The characteristics, natural density, experimental density, body mass, crawling speed, and experimental growth rate of the grazer species studied. The different characters in body size, crawling speed, and experimental growth rate indicated significant differences among the species (Tukey multiple comparison, *p* < 0.001, ANOVA, *p* < 0.001).

	*Glossosoma* sp.	*Micrasema quadriloba*	*Epeorus* sp.
Order	Trichoptera	Trichoptera	Ephemeroptera
Habit	Clinger	Clinger	Free clinger
Case	Turtle shell case	Small horned case	–
Natural density (ind. cm^−2^)	1.20 ± 0.22	0.15 ± 0.05	0.11 ± 0.12
Experimental density (ind. cm^−2^)	1.13	0.148	0.148
Experimental density (ind. /tile)	23	3	3
Body size (mg dry mass)	0.7 ± 0.1 a	0.2 ± 0.0 a	10.5 ± 3.6 b
Crawling speed (cm m^−1^)	1.1 ± 0.3 a	1.8 ± 0.9 a	43.4 ± 35.5 b
Growth rate (µg day^−1^)	16.72 ± 1.07 a	65.56 ± 97.7 a	72.81 ± 418.1 a

**Figure 4:**
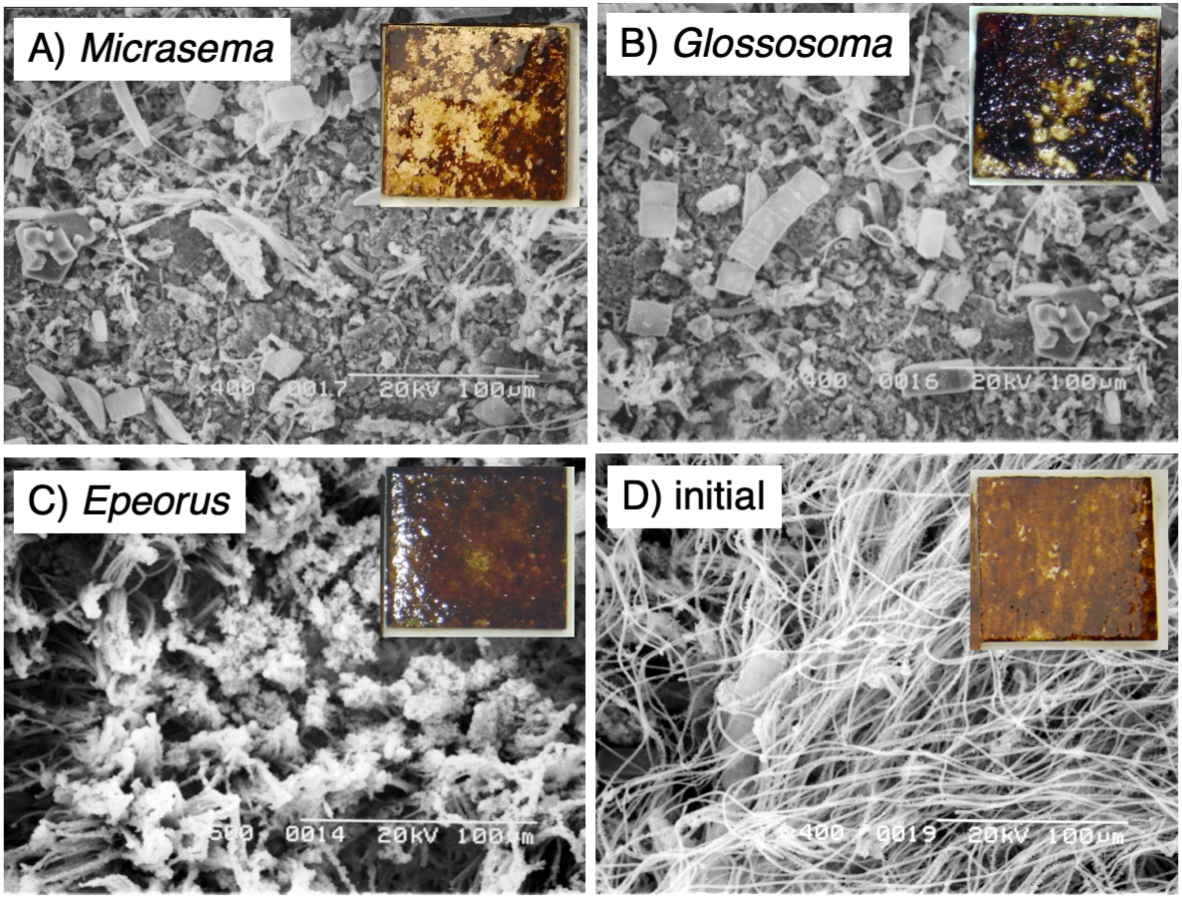
Scanning electron microscopy (SEM) images of the periphytic mats grown on the experimental tiles showing the grazed conditions for (A) *Micrasema quadriloba*, (B) *Glossosoma* sp., (C) *Epeorus* sp., and (D) the initial conditions. Magnitude and scale are shown in the images. The colored images are the experimental tiles with the initial and grazed conditions.

**Figure 5:**
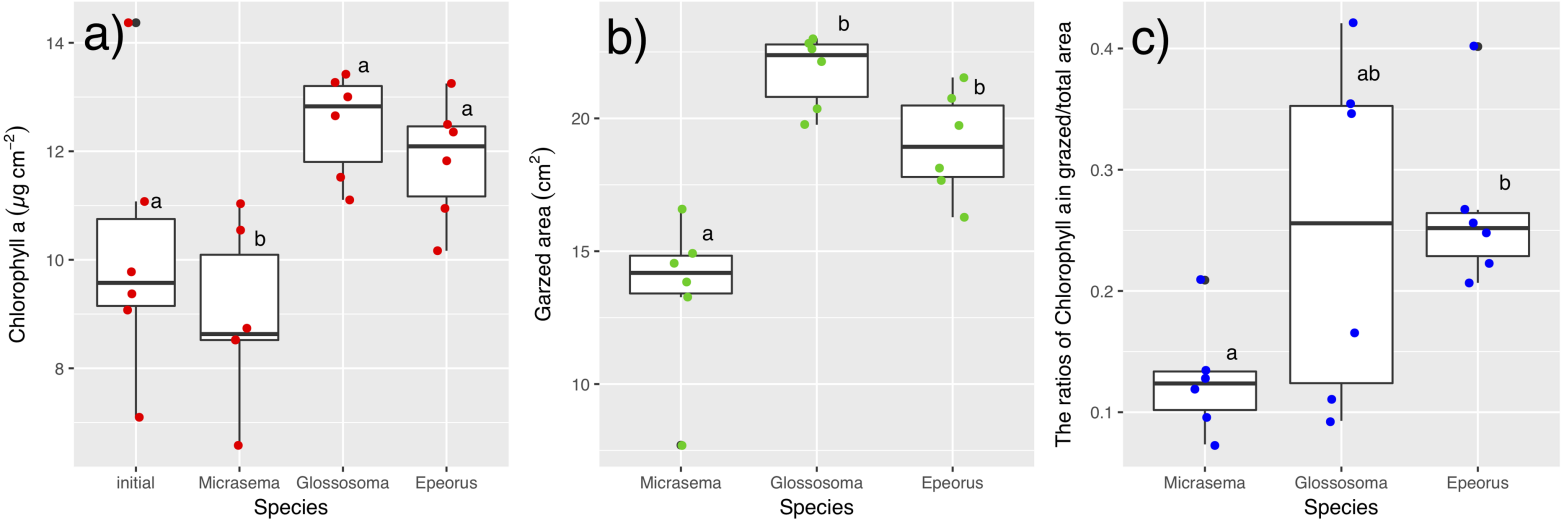
(A) Chlorophyll *a.* content, (B) grazed area, and (C) the ratios of chlorophyll *a* content in grazed/total periphyton area on tiles with different gazer species and controls. The bar in the box, upper and lower box edges, and error bar represent the median, ±25% quantile, and 1.5 ×±25% quantile, respectively. The dot points are each value. The different characters indicate significant differences found by Tukey post-hoc test (*P* < 0.05).

**Figure 6:**
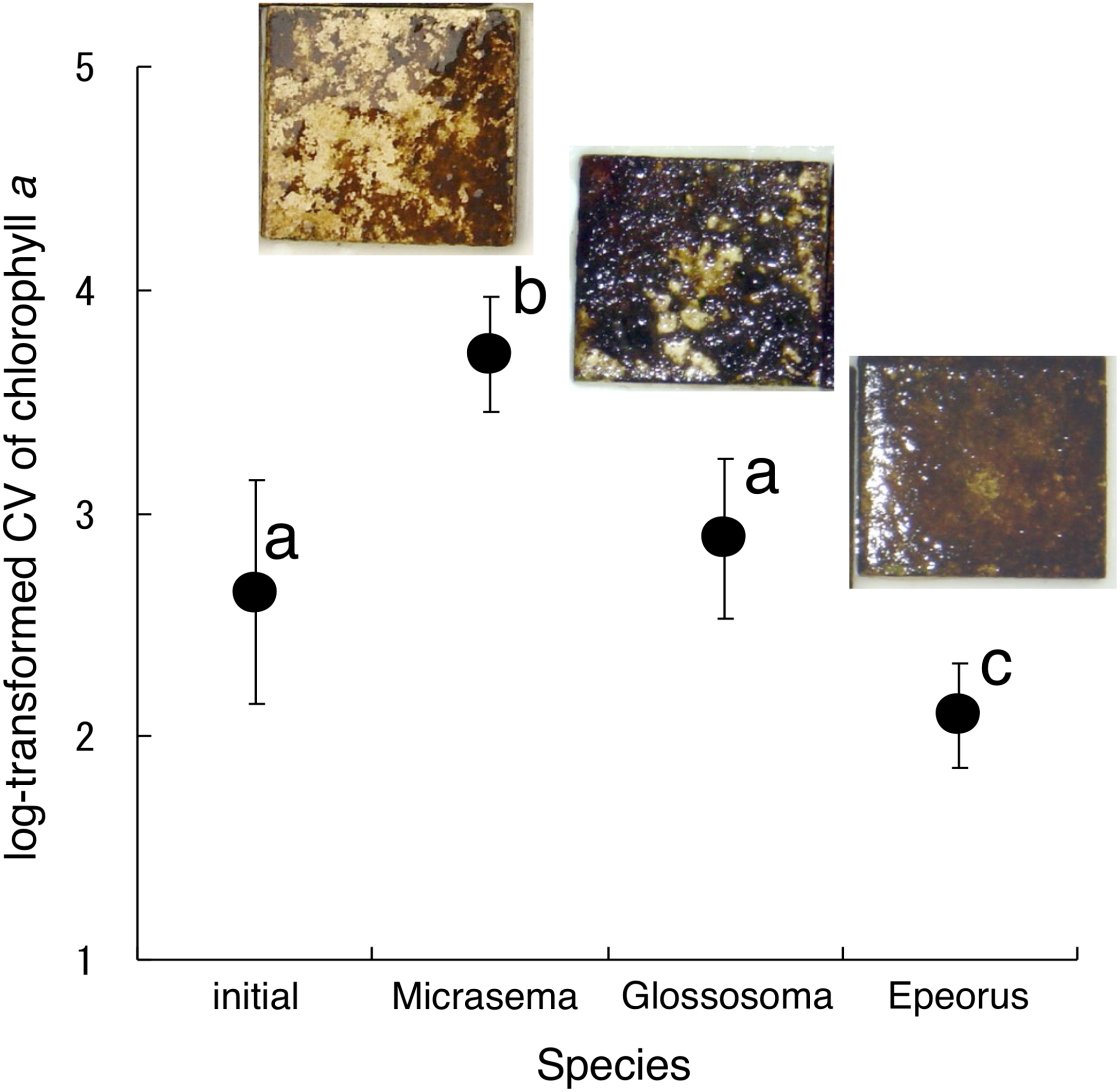
Log-transformed coefficient s of variation (CV) of chlorophyll *a* on the tiles with different gazer species. The error bars represent the standard deviation s (±1 SD). The photos on the error bars indicate each photo of the tiles with the grazer species. The different characters indicate significant differences found by Tukey’s post-hoc test (*P* < 0.05).

Different structures in the grazed periphyton were evident in the SEM images taken at the end of the experiment (Fig. 4). Ungrazed tiles appeared to be dominated by filamentous algae, whereas caddisfly-grazed tiles (*Glossosoma* sp. and *M. quadriloba*) were dominated by diatoms. We observed tangles of filamentous algae on *Epeorus*-grazed tiles, suggesting that *Epeorus* sp. might graze only on the upper layer of the periphyton, in contrast to the two caddisfly species. At the end of the enclosure experiment, the mat with *M. quadriloba* had the lowest Chl *a* content of the three grazer species (Fig. 5A, ANOVA; *F* = 5.72, *P* = 0.0054, Tukey; *P* < 0.05). Additionally, the grazing area with *M. quadriloba* was smaller than those with the other two species (Fig. 5B, ANOVA; *F* = 21.26, *P* < 0.001, Tukey; *P* < 0.001). The grazed/total area ratios of Chl *a* content in mats with *M. quadriloba* were lower than those with *Epeorus* sp., but were only marginally different to the mats with *Glossosoma* sp., possibly due to large variations of the grazed/total area ratios in *Glossosoma* sp. (Fig. 5C, ANOVA; *F* = 3.83, *P* = 0.045, Tukey; *P* = 0.054). The coefficients of variation (CV) of Chl *a* content in mats containing *M. quadriloba* was significantly higher than for both the other species and the initial control (Fig. 6, ANOVA; *F* = 3.81, *P* = 0.01, Tukey; *P* < 0.05).

## Discussion

We found that differences in the spatial heterogeneity of periphytic mats was the result of the grazing effects of three grazer species having different mouthpart morphologies and movement rates. Our results agree with those of Liess & Hillebrand (2004), who suggested that the spatial heterogeneity of producers resulted from grazer functional diversity. Moreover, our results showed large variability in the CV (spatial heterogeneity) associated with grazing effects. Thus, the observed effects on heterogeneity were dependent on the grazer feeding traits, explaining the contrasting results obtained in previous studies on algal variability (Liess & Hillebrand, 2004).

*Micrasema quadriloba*, with its scraping mouthpart morphology, had the strongest grazing effect on periphytic mats in terms of Chl *a* abundance and CV. *Micrasema quadriloba* also aggregated for group feeding in the experiment in Katano et al. (2007), which may have resulted in greater spatial heterogeneity in Chl *a* abundance with aggregated grazing impact (Katano et al., 2007). *Epeorus* sp. had the weakest effect on the spatial heterogeneity of Chl *a*, probably because of its brushing mouthpart morphology and rapid movement rate. *Glossosoma* sp. had only a moderate effect on the periphyton compared to the other two species, possibly because of its scraping mouthparts with large chisel-like mandibles; the presence of *Glossosoma* sp. also resulted in increased heterogeneity in the grazing/total area ratio, which may also have been due to grazing using chisel-like mandibles.

Natural selection is expected to foster traits, such as mouthpart morphology and feeding behavior that enhance grazer fitness (e.g., Feminella & Resh, 1990), especially when periphytic biomass is low. Reduced periphyton can impose strong selective pressure on grazer. For example, Feminella & Resh (1990) reported that a low periphytic biomass led to reduced growth and survival of trichopteran grazers. We observed that low periphyton availability resulted in an increase in the distance travelled by *Glossosoma* sp. larvae. This suggests that the larvae were traveling further to maximize their food intake (Kuhara, Nakano & Miyasaka, 2001), but grazer movement will increase energy costs and consequently lower fitness. Additionally, competition among grazers is generally very intense (McNeely, Finlay & Power, 2007), and grazers change their movements, drift behavior, and diel periodicity of their feeding activity accordingly (Kohler & McPeek, 1989; Kuhara, Nakano & Miyasaka, 2001; Miyasaka et al., 2003). Therefore, the consequences of their grazing performance and competition may have resulted from the adaptations of their feeding traits for periphyton.

We found that the periphytic mat structures were greatly modified by the grazer species, and that mat structures differed according to grazer mouthpart morphology, namely scraping (two caddisfly species) and brushing (*Epeorus* sp.)*.* Grazing depth is an important element for the formation of periphytic mats, as well as the community assemblages it hosts (such as bacteria and small invertebrates) and should be measured in future studies., Community structure largely depends on the structure of the periphytic mat (Pringle, 1990; Palmer, 1995; Wellnitz & Poff, 2006). Further studies are needed to evaluate the consequent effects of grazing on the entire periphytic community, including the effects on small consumers and bacteria.

## Conclusions

Our study revealed that differences in the spatial heterogeneity of periphytic mats were a consequence of the different functional traits of grazer species, such as mouthpart morphology and clinging activity. The largest effect on the spatial heterogeneity and abundance of the periphyton was from a caddisfly species with a scraping mouthpart morphology. The mayfly with brushing mouthpart morphology evoked weaker effects on the spatial heterogeneity of the periphytic mats. Here, we suggest that mouthpart morphology and movement rate largely influence the spatial heterogeneity of periphytic mats. Insect grazers are the primary consumers of periphyton in streams and serve as prey for consumers in higher trophic levels (such as fish and plecopterans) (Pringle, 1990; Palmer, 1995). Therefore, understanding the feeding traits and the feeding effects of primary consumers on periphyton and their fitness may be useful for understanding trophic interactions within the larger food web.

##  Supplemental Information

10.7717/peerj.6747/supp-1 Data S1 Supplemental materials for all raw data used in this study All raw data used in this study. The chlorophyll a (Chl-a) contents on the experimental tiles and the body size, speed and growth rates of the grazer species.

## Additional Information and Declarations

### Data Availability

The following information was supplied regarding data availability: The raw measurements are available in the Supplementary File.
